# Retinoic acid receptor beta and angiopoietin-like protein 1 are involved in the regulation of human androgen biosynthesis

**DOI:** 10.1038/srep10132

**Published:** 2015-05-13

**Authors:** Sameer S. Udhane, Amit V. Pandey, Gaby Hofer, Primus E. Mullis, Christa E. Flück

**Affiliations:** 1 Pediatric Endocrinology and Diabetology, Department of Pediatrics, University Children’s Hospital, Inselspital; 2 The Department of Clinical Research, University of Bern, 3010 Bern, Switzerland

## Abstract

Androgens are essential for sexual development and reproduction. However, androgen regulation in health and disease is poorly understood. We showed that human adrenocortical H295R cells grown under starvation conditions acquire a hyperandrogenic steroid profile with changes in steroid metabolizing enzymes HSD3B2 and CYP17A1 essential for androgen production. Here we studied the regulatory mechanisms underlying androgen production in starved H295R cells. Microarray expression profiling of normal versus starved H295R cells revealed fourteen differentially expressed genes; HSD3B2, HSD3B1, CYP21A2, RARB, ASS1, CFI, ASCL1 and ENC1 play a role in steroid and energy metabolism and ANGPTL1, PLK2, DUSP6, DUSP10 and FREM2 are involved in signal transduction. We discovered two new gene networks around RARB and ANGPTL1, and show how they regulate androgen biosynthesis. Transcription factor RARB stimulated the promoters of genes involved in androgen production (StAR, CYP17A1 and HSD3B2) and enhanced androstenedione production. For HSD3B2 regulation RARB worked in cooperation with Nur77. Secretory protein ANGPTL1 modulated CYP17A1 and DUSP6 expression by inducing ERK1/2 phosphorylation. By contrast, our studies revealed no evidence for hormones or cell cycle involvement in regulating androgen biosynthesis. In summary, these studies establish a firm role for RARB and ANGPTL1 in the regulation of androgen production in H295R cells.

Steroid hormones are essential for mammalian life and reproduction. They are mainly synthesized in endocrine organs such as the adrenal glands, gonads and the placenta. Based on their biological function(s) steroid hormones are classified in three main groups, mineralocorticoids, glucocorticoids and sex steroids (androgens and estrogen). Sex steroids are essential for both male and female sexual development and reproduction. Precursors of androgens are also produced in the fetal adrenals as well as the zona reticularis (ZR) of the adult adrenal cortex. The regulatory system controlling the development of the ZR and the androgen production of the ZR are largely unknown. However, it is known that the adrenocorticotropic hormone (ACTH) and its signaling network, which regulate glucocorticoid production in the zona fasciculata (ZF) of the adrenal cortex, play a co-regulatory role for androgen production^1^. By contrast, estrogen and testosterone production in the ovary and testis are regulated through the gonadotropin-releasing hormone (GnRH) of the hypothalamus and the luteinizing hormone (LH) and the follicle-stimulating hormone (FSH) of the pituitary gland^2^.

Cholesterol, the building block of all steroid hormones, is transported to the mitochondria through the help of the steroidogenic acute regulatory protein (StAR). At the inner mitochondrial membrane the side-chain cleavage system (CYP11A1-FDX-FDXR) catalyzes the conversion of cholesterol to pregnenolone, which is needed for the production of all steroids. Steroid biosynthesis then proceeds further via a series of enzymatic reactions which involves the enzymes cytochrome P450c17 (encoded by *CYP17A1*) and 3β-hydroxysteroid dehydrogenase II (3βHSDII) (encoded by *HSD3B2*) that are essential for the production of all sex steroids.

In humans, adrenarche is the event of functional activation of the ZR of the adrenal cortex which occurs at around 6 to 8 years of age. It consists of an increased production and secretion of adrenal androgens, namely the 19-carbon (C19) steroids dehydroepiandrosterone (DHEA) and dehydroepiandrosterone sulfate (DHEAS). At adrenarche, the increased androgen production in the ZR is characterized by a decrease in HSD3B2, an increase in CYP17A1-17,20-lyase activity as well as an increase in cytochrome b5 (CYB5), P450 oxidoreductase (POR) and type 2 dehydroepiandrosterone sulfotransferase (SULT2A1)^3^,^4^. Alterations in androgen production pose health problems^5^. The hyperandrogenic disorder of polycystic ovary syndrome (PCOS) is the most common endocrine disorder in women associated with infertility and metabolic consequences. In PCOS, an increased expression of HSD3B2 and CYP17A1 was found in the ovarian theca cells suggesting that these enzymes play an important role in this syndrome^6^. However, the detailed molecular mechanisms underlying the increased androgen production remain obscure, making a targeted treatment impossible.

The adrenocortical carcinoma NCI-H295 cell lines are an established model to study the regulation of steroidogenesis as primary cell culture or small animal models do not exist^7^,^8^,^9^,^10^. The H295R cells synthesize all steroids, which are normally produced in the three layers of the human adrenal cortex. We have shown that specially subcloned H295R cells are a suitable model for studying androgen biosynthesis^11^. We found that H295R cells have lower 3βHSDII and higher 17,20-lyase activity, resembling the steroid profile of the androgen producing ZR of the human adrenal cortex^11^. We also found that the steroid profile of H295R cells differs with various cell growth conditions^12^. A serum-free (starvation) growth condition shifts steroidogenesis of H295R cells towards androgen production. Serum starvation leads to an increase in CYP17A1 17,20-lyase activity and a decrease in HSD3B2 expression and activity^12^. In general, serum starvation is recognized as a model to study cell cycle, hormonal regulations and metabolic processes^13^,^14^,^15^,^16^,^17^,^18^. Several studies have illustrated that serum-free culturing conditions impact on several intracellular signaling pathways; for example AMP-activated protein kinase (AMPK) in skeletal muscle cells^14^, Mitogen-activated protein kinases (MAPKs) pathway in cardiac fibroblasts^19^ and protein kinase B (AKT/PKB) in glioma cells as well as in embryonic fibroblast cells^20^,^21^. Previously, we investigated the role of AMPK signaling on androgen production in adrenal H295R cells but found no obvious connection^22^.

Multiple signaling pathways regulate steroidogenesis^23^. In general, adrenal androgen biosynthesis is coregulated by the peptide hormone ACTH via the cyclic adenosine monophosphate (cAMP)/cAMP-dependent protein kinase A (PKA) pathway^1^,^3^,^23^. Additional pathways have been reported^23^, but it is still believed that the main regulators have yet to be determined. The interplay between the cAMP/PKA and the phosphoinositide 3’-OH kinase (PI3K/AKT) signaling for the regulation of steroidogenesis is described in rat granulosa cells^24^. MAPKs regulate many intracellular events^25^. Recent studies have demonstrated that in PCOS theca cells reduced levels of activated mitogen-activated protein kinase (MEK1/2) and extracellular signal-regulated kinase 1/2 (ERK1/2) correlate with increased androgen production^26^. These findings implicate alterations in the MAPK pathway in the pathogenesis of excessive ovarian androgen production in PCOS. Most recently MAPK member 14 (MAPK14), also called p38α, has been shown to phosphorylate P450c17 in a fashion that confers increased 17,20-lyase activity which is essential for the production of androgens^27^.

In this study we investigated the gene expression profiles and specific signaling pathways in androgen producing H295R cells to obtain further insight into androgen regulation in humans. We used the previously described H295R cell starvation model which causes a shift in the steroid profile towards androgen production and produces a differential gene expression profile of a hyperandrogenic state. We used gene expression profiling, bioinformatics analysis and signal transduction analysis to identify the genes involved in the regulatory network of androgen biosynthesis. Interestingly, we found only 14 genes differentially expressed. Among them retinoic acid receptor beta (RARB) and angiopoietin-like protein 1 (ANGPTL1) led us to novel pathways involved in the regulation of androgens.

## Results

### Serum starvation enhances androgen production in H295R and MA-10 cells

**Figure 1:**
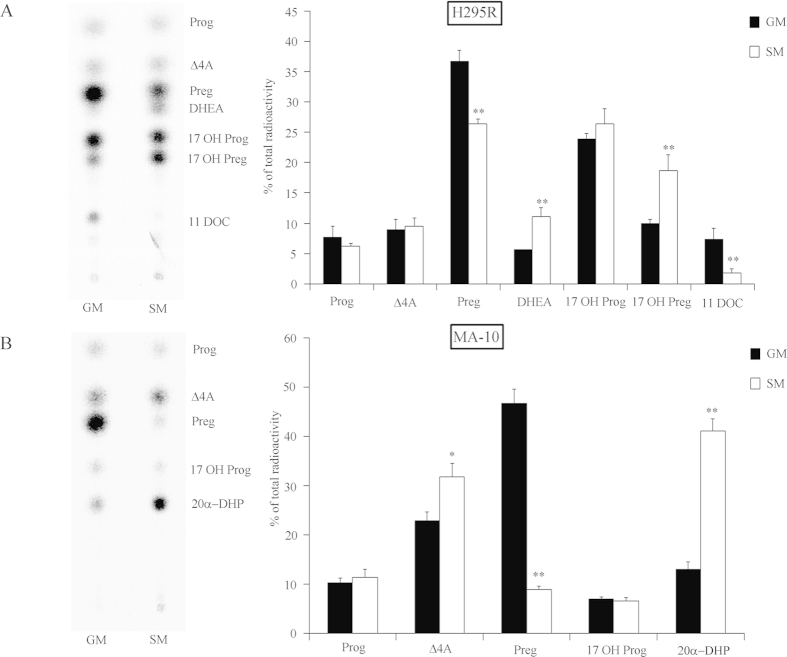
Serum starvation enhances androgen production in human adrenal H295R and mouse Leydig MA-10 cells. Steroid profiles of H295R and MA-10 cells grown under starvation growth condition (SM) for 48 h were assessed and compared to normal growth conditions (GM). Steroid production was labeled with [^3^H] pregnenolone for 90 min. Steroids were extracted and resolved by TLC. A representative TLC is shown on the left and quantitative analysis of four independent experiments on the right. (**A**) Steroid profiling of H295R cells in GM vs SM conditions. (**B**) Steroid profiling of MA-10 cells in GM vs SM conditions. Data are the mean ± SD of three to four independent experiments. * p < 0.05, ** p < 0.01. Prog, progesterone; Δ4A, androstenedione; Preg, pregnenolone; 17OH Preg, 17α-hydroxypregnenolone; 17OH Prog, 17OHP; 11DOC, 11-deoxycortisol, 20α-DHP, 20α-dihydro-progesterone.

We found that starvation in human adrenal H295R cells induced androgen production (Fig. 1A)^12^. We then looked at effects of starvation on steroidogenesis of mouse Leydig cells to check for a common regulatory mechanism, which would not be tissue-specific and/or species-specific. We starved mouse Leydig MA-10 cells similar to H295R cells and assessed their steroid profile using labeled pregnenolone (Fig. 1B). The testosterone biosynthesis pathway varies between species, where humans and rabbits predominantly use the Δ^5^ pathway to produce androgens from pregnenolone^28^, whereas rats and mice use the Δ^4^ pathway^28^,^29^. However, MA-10 cells do not produce testosterone but predominantly 20α-dihydroprogesterone (20α-DHP) and express little CYP17A1^30^,^31^,^32^. Starvation of MA-10 cells for 48 hours significantly increased the conversion of pregnenolone to androstenedione and 20α-DHP (Fig. 1B). Thus, starvation was found to induce steroidogenesis in humans and mice and resulted in higher androgen production in both the adrenal cortex and the testis.

### The androgen shift observed in starved H295R cells is not mediated by hormones

**Figure 2:**
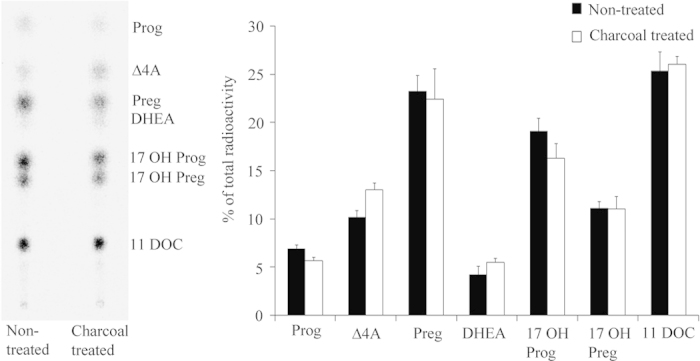
Effect of hormones on the steroid profile of H295R cells. H295R cells are usually grown in DMEM/Ham’s F-12 medium enriched with NU-I serum (according to ATCC guidelines). To remove hormones we stripped NU-I serum by charcoal treatment (see Methods). H295R cells were then grown in normal growth medium with charcoal-stripped NU-I serum medium and the steroid profile was assessed after 48 hours. Steroid production was labeled with [^3^H] pregnenolone for 90 min and extracted steroids were resolved by TLC. A representative TLC is shown on the left and the quantitative analysis of steroids showing no change is shown on the right. Data are the mean ± SEM of two independent experiments. Prog, progesterone; Δ4A, androstenedione; Preg, pregnenolone; 17OH Preg, 17α-hydroxypregnenolone; 17OH Prog, 17OHP; 11DOC, 11-deoxycortisol.

Serum used in culture media contains many substances including growth factors, cytokines, fatty acids, lipids, vitamins, trace elements, carbohydrates and hormones^33^. Since serum withdrawal enhanced androgen production in H295R cells^12^, we investigated whether hormones might be responsible for these alterations in the steroid production. Therefore, we removed hormones from the H295R NU-I serum by charcoal treatment as described previously^34^. Charcoal stripping of serum removes hormones such as steroids, peptide hormones and thyroid hormone^34^,^35^. To confirm effectiveness of the NU-I serum stripping, we measured testosterone concentrations before and after charcoal stripping with a radioimmunoassay (Beckman Coulter, RIA Testosterone kit). After charcoal stripping, testosterone was no longer detectable in NU-I serum. The steroid production was then assessed in H295R cells cultured in normal growth medium supplemented with full NU-I serum or medium supplemented with charcoal stripped NU-I serum. Steroid conversion was measured using radiolabeled pregnenolone and TLC readout (Fig. 2). Charcoal stripping did not change the steroid profile of H295R cells. This indicates that hormones in the serum most likely are not responsible for the alterations of the steroid production observed in H295R cells when grown without serum.

### The starvation effect on steroidogenesis does not depend on changes of the cell cycle

**Figure 3:**
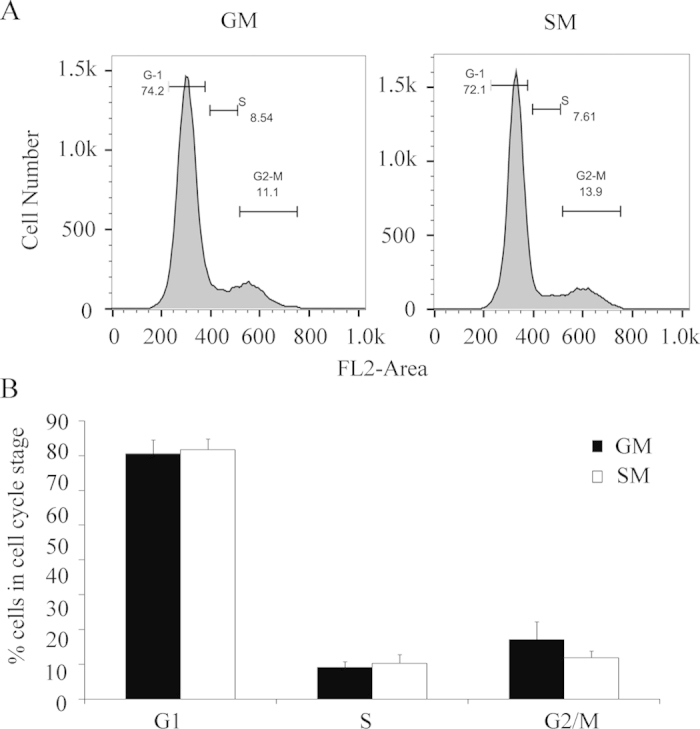
Effect of serum starvation on the cell cycle in H295R cells. H295R cells were grown in normal growth medium or serum free (starvation) medium for 48 hours. Cells were harvested and the DNA content was analyzed by propidium iodide staining to assess cell cycle characteristics by FACS. (**A**) Cell cycle profile of H295R cells in normal growth (GM) and serum starvation (SM) medium. FL2-Area represents DNA content (**B**) Quantitative analysis of three independent experiments by specific cycle stages in percentage. Error bars show the mean ± SD.

The serum starvation model has been extensively used for studying the cell cycle synchronization^36^,^37^. Therefore, we investigated the effects of serum starvation on the cell cycle regulation in H295R cells using FACS analysis. However, FACS analysis showed that 48 hours of serum starvation did not significantly affect the cell cycle regulation in H295R cells, suggesting that starvation induced androgen production probably does not depend on cell cycle mechanisms (Fig. 3).

### Identification of differentially expressed genes in starved H295R cells

To characterize the gene expression profile of H295R cells cultured under serum-starved conditions, we performed microarray studies using the GeneChip Human Gene 1.0 ST arrays. For analysis, the *P* values were adjusted for multiple testing with Benjamini and Hochberg’s method to control for a false discovery rate (FDR). Probe sets showing at least a 2-fold change and a FDR < 0.05 were considered significant. We identified 14 genes with a significantly altered (>2.0 fold) expression profile, when comparing starved with control H295R cells (Table 1). The identified genes and their putative biological functions are given in Table 2. Serum starvation reduced the expression of steroidogenic genes 21-hydroxylase (CYP21A2), HSD3B1 and HSD3B2. In the signal transduction pathway polo like kinase 2 (PLK2), dual specificity phosphatase 6 and 10 (DUSP6 and DUSP10), FRAS1 related extracellular matrix protein 2 (FREM2) and ANGPTL1 had a reduced expression under starvation conditions.

**Figure 4:**
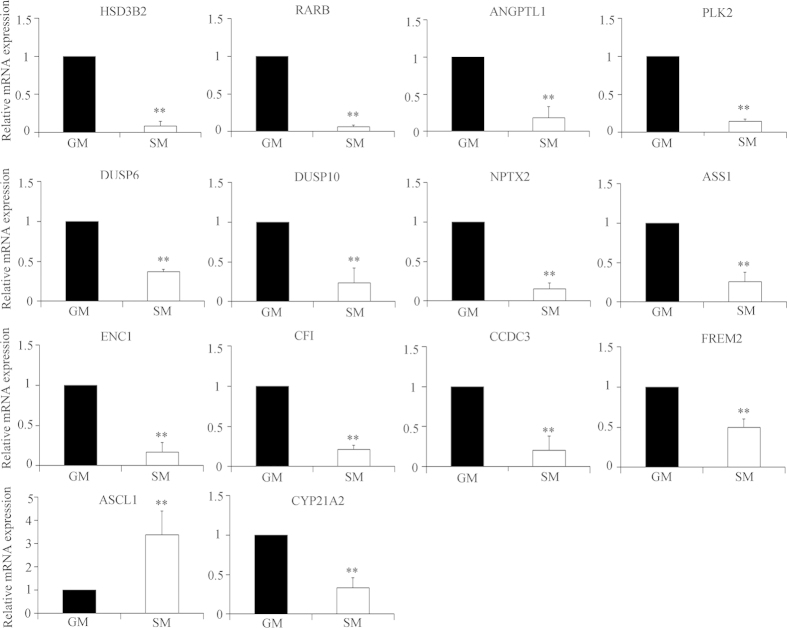
Gene expression profiling for starved H295R cells by qRT-PCR. Validation of gene expression data obtained from microarrays was performed by qRT-PCR. Total RNA was isolated from H295R cells grown in normal (GM) or starvation medium (SM). Expression of the genes identified by the microarrays with a significance level of fold change >2 was analyzed by SYBR Green based qRT-PCR. The cyclophilin A gene was used as internal control for data normalization. Analysis of relative gene expression values was performed by the 2^−ΔΔCt^ method. Summaries of qRT-PCR results of three independent experiments (mean ± SD) are shown. * p < 0.05, ** p < 0.01.

Hierarchical clustering was applied to the gene expression data using complete linkage algorithm in Cluster 3.0 software and visualized by the JTreeView software. A heat map for the microarray data was drawn showing the gene expression profiles of H295R cells cultured under normal growth and starvation conditions (Supplementary Figure S1). To confirm the microarray findings, we performed SYBER Green based qRT-PCR analysis of selected 14 transcripts (Fig. 4). All genes which were significantly up- or down-regulated under starvation conditions by microarray analysis of >2.0 fold (p < 0.05), were confirmed by specific qRT-PCRs. In addition, the CYP21A2 transcript which was found to be regulated at a level of >1.5 fold (p < 0.05) in the microarray (Supplementary Table S2), was also tested and confirmed by qRT-PCR.

### Analysis of differentially expressed genes in starved H295R cells

**Figure 5:**
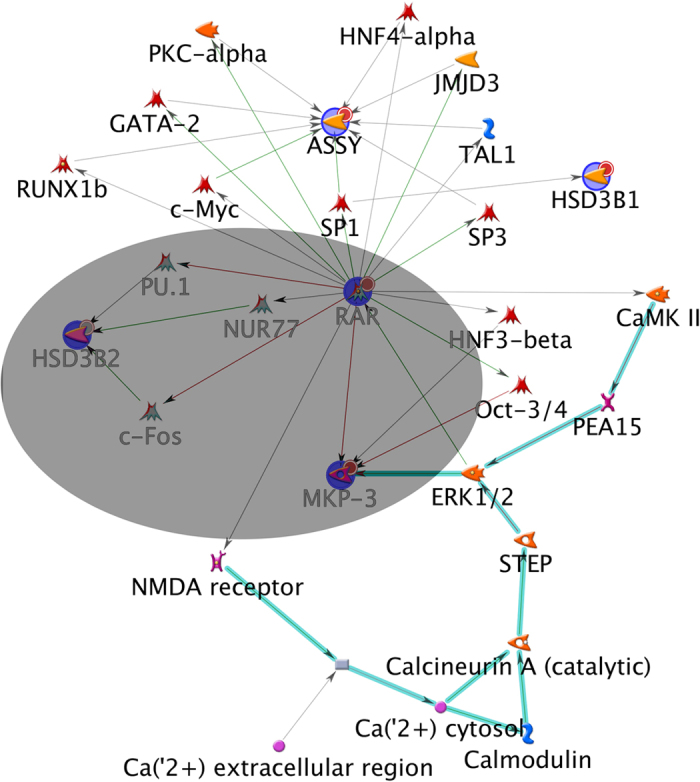
Network analysis of differentially expressed genes in starvation conditions. GeneGo Metacore software was used to analyze the microarray data for common gene networks between significantly changed genes. Five genes were found in a possible common network, namely HSD3B2, RAR, ASSY, MKP3 (DUSP6) and HSD3B1. The center of this network consists of the RAR gene which links to HSD3B2 through Nur77, and MKP3 seems in a relationship with RAR and ERK1/2. The core of the identified network is highlighted in grey. Only down-regulated genes were found in this network and they are highlighted in blue and marked with small red circles. Green lines show activating relationships; red lines show inhibition; gray lines show relationships of unknown quality; turquoise bold lines connect members of the RAS superfamily.

To check whether the differentially expressed genes from the starved, hyperandrogenic H295R cells form a network for androgen regulation, microarray data were imported to GeneGo Metacore analysis software for gene set enrichment analysis. This program is able to recognize gene networks on the basis of previously reported functional interactions (www.genego.com). Analysis of our data revealed a network in disease biomarker processes which linked 5 out of the 14 genes, namely DUSP6 also called mitogen kinase phosphatase 3 (MKP-3), HSD3B1, argininosuccinate synthase 1 (ASS1), HSD3B2 and retinoic acid receptor (RAR) (Fig. 5). These five genes were significantly down regulated under starvation conditions. RAR was the center node of this network which linked to several other factors; but most important in our context, RAR linked to Nur77 and HSD3B2. This network suggests that RARB may regulate HSD3B2 through Nur77.

We then used DAVID software to study the possible functional role of differentially expressed genes under starvation conditions^38^. We found that the identified genes were involved in highly selected biological processes (Table 2). Most of the genes seemed to play a role in energy metabolism; other genes showed possible involvement in signal transduction, especially the mitogen-activated protein kinase kinase kinase (MAPKKK) cascade, and three genes were involved in steroidogenesis (Table 2).

### Regulation of steroidogenesis by nuclear receptor RARB

The specific role of the RARB in steroidogenesis is unknown, although RARB seems to be expressed in normal human adrenal and gonadal tissues (GEO GDS3113/109692/RARB). RARs are nuclear receptors which are activated upon binding of ligands such as all-trans-retinoic acid (ATRA). ATRA is an activated metabolite of retinol (vitamin A) which has been described to regulate the transcription of steroidogenic genes expressed in the adrenal cortex, the testis and the ovary^39^,^40^,^41^. Studies in rats revealed that retinol deficiency decreased HSD3B2 activity *in* and *ex vivo* in adrenals, testes and ovaries; and that this effect was rescued by retinol or RA supplementation^42^. In testis, retinol may be metabolized to active (AT)RA to act as ligand on its receptor(s)^43^. Distinct genes (α, β, and γ) encode the different retinoic acid receptors (RARs). RARs control specific gene expressions in a ligand-dependent manner, and belong to the nuclear receptor 1 B (NR1B) subtype of the nuclear receptor superfamily. They play a role in development, cell growth and survival, vision, spermatogenesis, immune function, and neural functions^44^.

**Figure 6:**
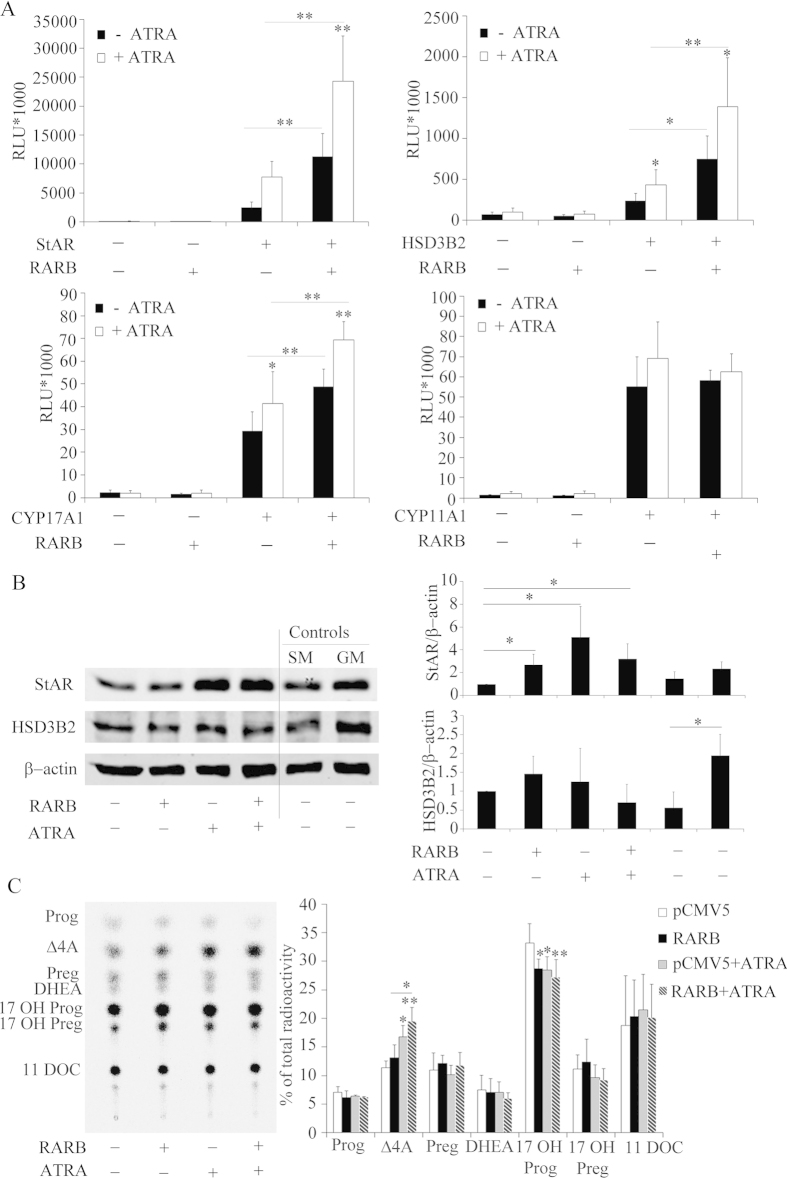
Role of the nuclear transcription factor RARB in altering the expression of genes involved in steroidogenesis. (**A**) H295R cells were transfected with the human −1.05 kb HSD3B2, −1.3 kb StAR and −3.7 kb CYP17A1, -2327 bp CYP11A1 promoter constructs along with an expression vector for the RARB transcription factor. Transfection medium was changed after 6 h and cells were cultivated in serum free medium for 24 h. Thereafter, cells were grown in the presence or absence of ATRA in serum-free medium for another 24 h. Promoter activation was assessed by the dual luciferase assay (Promega) using pRL-TK as internal control. Data are expressed in Relative Light Units (RLU). Error bars show the mean ± SD of three independent experiments. * p < 0.05, ** p < 0.01. (**B**) Effect of RARB overexpression in absence or presence of ATRA was studied with respect to endogenous StAR and HSD3B2 expression. H295R cells were transiently transfected with RARB or the empty vector for control. Transfected cells were optionally treated with ATRA for 24 h in starvation medium. Finally, the effect of RARB on StAR and HSD3B2 was assessed by performing specific Western blot analyses. A representative blot is shown on the left and quantitative analysis on the right. Data are the mean ± SD of three independent experiments. (**C**) Effect of RARB overexpression and ATRA treatment on H295R steroid production. H295R cells were transiently transfected with RARB or the empty vector for control. Transfected cells were optionally treated with 1 μM ATRA for 24 h in starvation medium. Steroid production was labeled with [^3^H] pregnenolone for 90 min and extracted steroids were resolved by TLC. A representative TLC is shown on the left and the quantitative analysis of steroids shown on the right. Data are the mean ± SEM of three independent experiments. * p < 0.05, ** p < 0.01. Prog, progesterone; Δ4A, androstenedione; Preg, pregnenolone; 17OH Preg, 17α-hydroxypregnenolone; 17OH Prog, 17OHP; 11DOC, 11-deoxycortisol.

After identifying RARB as a possible regulator of HSD3B2 (Fig. 5), we investigated its role in regulating transcription of steroid genes in the absence or presence of the ATRA ligand. We therefore transfected H295R cells with promoter constructs of four steroid genes (−1.05 kb HSD3B2, −1.3 kb StAR, −3.7 kb CYP17A1 and −2.23 kb CYP11A1) and a RARB expression vector with and without ATRA treatment in starved conditions (Fig. 6A). RARB overexpression in H295R cells was found to activate the promoters of StAR, HSD3B2 and CYP17A1, but not of CYP11A1. In the presence of the ATRA ligand, this activation became more pronounced for the RARB regulated promoters, and remained unaltered for CYP11A1 (Fig. 6A). To confirm these results we studied the effect of RARB overexpression on endogenous gene expression of StAR and HSD3B2 using Western blots (Fig. 6B). Overexpression of RARB increased StAR expression and addition of an ATRA ligand to starvation medium increased StAR expression with and without RARB overexpression (Fig. 6B), suggesting that ATRA might also work through endogenous RARB, RARα or RXRα^45^. In contrast, RARB overexpression or ATRA ligand treatment did not change HSD3B2 expression (Fig. 6B). Therefore, RARB might not directly regulate HSD3B2 or depend on another partner.

Further, we investigated whether an effect of RARB and ATRA might be seen on the steroid profile of H295R cells. Starved H295R cells were transfected with RARB in presence and absence of ATRA treatment and the steroid profile was assessed using pregnenolone labeling (Fig. 6C). Overexpression of both RARB and ATRA treatment increased androstenedione production and decreased 17-hydroxyprogesterone production; and this effect was most pronounced when combining RARB with ATRA. Calculations for an effect of RARB and ATRA on HSD3B2 activity revealed a significant increase (data not shown) confirming that RARB has a role in regulating adrenal androgen production.

### RARB and Nur77 co-operate to regulate HSD3B2

Since overexpression of RARB in starvation did not change endogenous HSD3B2 expression, we hypothesized that RARB required an interacting partner to act on HSD3B2. Our network studies suggested that RAR was a possible regulator of HSD3B2 via Nur77 (Fig. 5). In the Biological General Repository for Interaction Datasets (BioGRID; www.thebiogrid.org), we found evidence that RARB interacts with the nuclear receptor 4A2 (NR4A2), which belongs to the nuclear receptor 4A family that also includes Nur77 as a member. Furthermore, RARB has been shown to interact with Nur77 in human hepatocellular carcinoma cells and thus leads to apoptosis^46^. Also, Nur77 is an essential transcription factor for HSD3B2 expression^47^,^48^.

**Figure 7:**
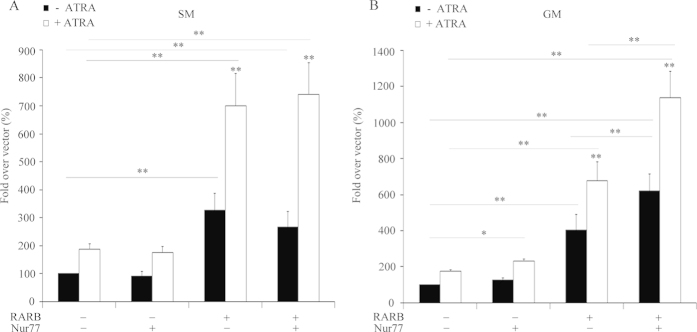
RARB and Nur77 co-operation regulate HSD3B2 in H295R cells. H295R cells were transfected with the human −1.05 kb HSD3B2 promoter constructs along with an expression vector RARB, Nur77 and in combination of RARB/Nur77. Transfection medium was changed after 6 h and cells were cultivated in either serum free or in normal growth medium for 24 h. Thereafter, cells were grown in the presence or absence of ATRA for another 24 h. (**A**) RARB and Nur77 co-operation study under serum starvation condition. (**B**) RARB and Nur77 co-operation study under normal growth condition. Promoter activation was assessed by the dual luciferase assay (Promega) using pRL-TK as internal control. Data are expressed in Relative Light Units (RLU). Error bars show the mean ± SD of four independent experiments. * p < 0.05, ** p < 0.01.

We therefore tested the possible cooperation between RARB and Nur77 for HSD3B2 expression. We transfected H295R cells with the −1050 bp HSD3B2 promoter reporter and the expression vectors for the transcription factors RARB and Nur77. RARB was found to activate the HSD3B2 promoter both in the presence and absence of ATRA (Fig. 7A), which was in line with earlier experiments (Fig. 6A). Nur77 alone did not enhance the HSD3B2 promoter activity (Fig. 7A). This result suggests that Nur77 alone is not sufficient to regulate HSD3B2 promoter activity in starvation. Based on our and reported data we postulated that Nur77 action itself depended on RARB expression^46^. Therefore, we investigated the cooperation of RARB and Nur77 on HSD3B2 in H295R cells cultivated in normal growth medium. Under these conditions Nur77 enhanced HSD3B2 promoter activity in the presence of the ATRA (Fig. 7B). Similar to starvation conditions, the combined stimulation with RARB and Nur77 increased the HSD3B2 promoter activity under normal growth conditions. Thus, RARB is essential for Nur77’s regulation of HSD3B2.

### ANGPTL1 modulates CYP17A1 expression through ERK1/2

Microarray data revealed that ANGPTL1 was significantly down-regulated under starvation conditions (Table 1). ANGPTL1 is known to be a potent regulator of angiogenesis or anti-angiogenesis depending on the cell context^49^,^50^. It also exhibits anti-apoptotic activity in endothelial cells by stimulating phosphorylation of ERK1/2 and AKT^50^. ANGPTL1 is known to participate in signal transduction via integrin^51^ and tyrosine kinase signaling pathways^49^,^52^. It has been shown that ANGPTL1 affects the phosphorylation of intracellular signaling kinases in cancer cells, like focal adhesion kinase (FAK), AKT and ERK1/2^51^. A recent study demonstrated that reduced levels of activated MEK1/2 and ERK1/2 in PCOS theca cells are correlated with increased CYP17A1 mRNA and DHEA synthesis^26^. Based on this knowledge we hypothesized that ANGPTL1 might affect androgen biosynthesis through intracellular ERK and AKT signaling.

**Figure 8:**
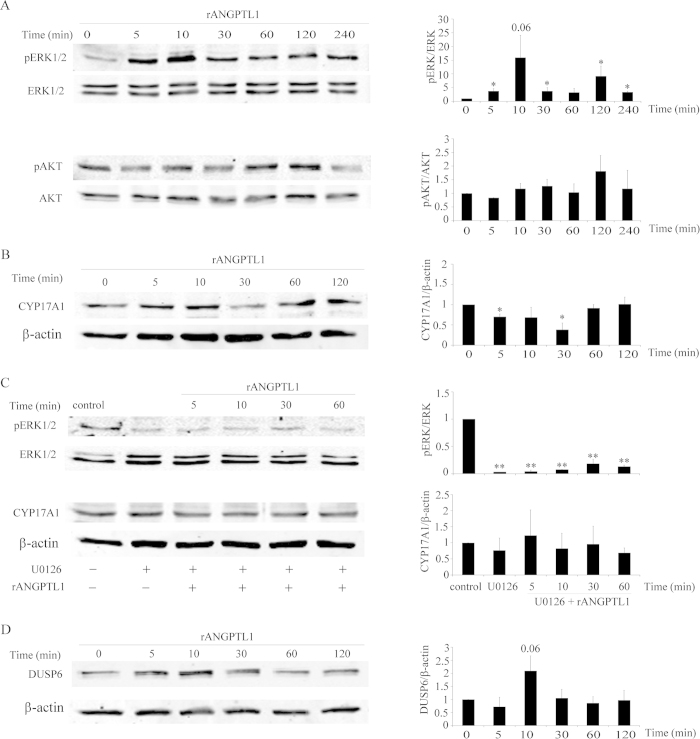
ANGPTL1 suppresses CYP17A1 expression through inducing ERK1/2 phosphorylation in starved H295R cells. (**A**) Effect of rANGPTL1 protein on ERK and AKT kinase phosphorylation: Western blot analysis was performed on starved H295R cells treated with 50 ng/ml rANGPTL1 for 0-4 h to study the changes in phosphorylation of ERK1/2 and AKT kinases. Western blot was performed on total protein preparations from treated starved H295R cells. Levels of total ERK and AKT were also analyzed. (**B**) Effect of rANGPTL1 treatment on steroidogenic CYP17A1 expression. Western blot analysis showed CYP17A1 expression was suppressed under treatment with rANGPTL1 in 30 min. We used β-actin as loading control. (**C**) The effect of inhibiting MEK/ERK1/2 phosphorylation on CYP17A1 expression in ANGPTL1 treated starved H295R cells. Cells were starved for 48 hrs and treated or not with rANGPTL1. When indicated, cells were pretreated with 10 μM U0126 for 30 min before rANGPTL1 treatment. Cells were lysed and levels of CYP17A1 and pERK1/2 were analyzed by Western blotting. Levels of total ERK were taken as a loading control. (**D**) Effect of rANGPTL1 on DUSP6 expression. Levels of DUSP6 were assessed by Western blotting. Representative blots are shown on the left and the quantitative analysis of blots is shown on the right. Data are the mean ± SEM of two or three independent experiments. * p < 0.05, ** p < 0.01.

We used recombinant ANGPTL1 protein and assessed its effect on ERK1/2 and AKT phosphorylation in starved H295R cells. As expected, we found low phosphorylation of ERK1/2 in starved H295R cells. ANGPTL1 treatment increased ERK1/2 phosphorylation significantly within 10 min (Fig. 8A). No alterations were found in AKT phosphorylation after ANGPTL1 treatment (Fig. 8A). We then determined whether ERK1/2 phosphorylation through ANGPTL1 might change CYP17A1 expression in starved H295R cells. ANGPTL1 decreased CYP17A1 expression within 30 min (Fig. 8B). To confirm our findings, MEK/ERK1/2 signaling was inactivated, hypothesizing that the effect of ANGPTL1 on CYP17A1 expression would be abolished. For this we used the specific MEK/ERK 1/2 phosphorylation inhibitor U0126. Treatment of starved H295R cells with U0126 reduced ERK1/2 phosphorylation dramatically, and ANGPTL1 was not able to counteract this phosphorylation blockade (Fig. 8C). As expected, inhibition of MEK/ERK1/2 phosphorylation allowed for unsuppressed CYP17A1 expression (Fig. 8C). These results confirm that ANGPTL1 may decrease CYP17A1 expression through ERK1/2 phosphorylation.

**Figure 9:**
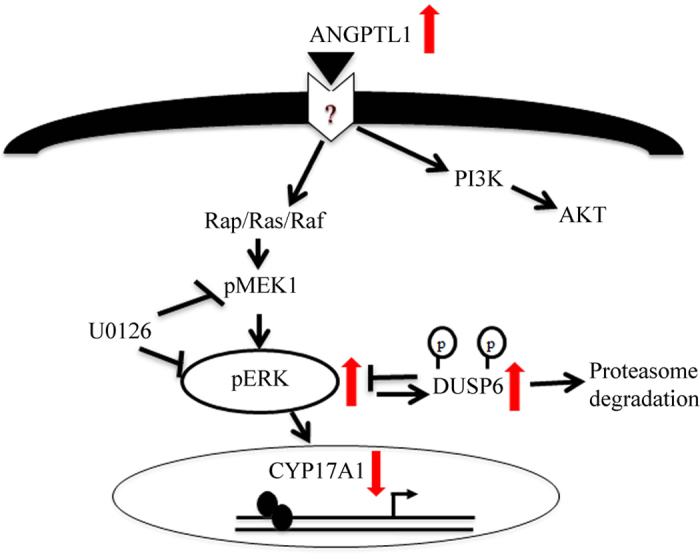
Scheme of suggested regulation of androgen production by ANGPTL1 in H295R cells based on presented findings (arrows) and information from literature 49,50,51,52,56. ANGPTL1 enhances ERK1/2 phosphorylation and thereby modulates CYP17A1 expression. ANGPTL1 also stimulates DUSP6 expression. DUSP6 and ERK1/2 regulate each other; while DUSP6 may dephosphorylate ERK1/2, phospho-ERKs phosphorylate DUSP6 prompting its degradation.

In addition, our microarray data also indicated that starvation significantly decreased DUSP6 expression in H295R cells. DUSP6 is a cytoplasmic protein that is also recognized as MAPK phosphatase 3 (MKP-3)^53^. It is known to dephosphorylate the specific cytosolic ERK1/2^54^. MAPK phosphorylation and thus activity is a reversible process which is controlled by MAPK kinases and phosphatases (MKPs) activity^55^. MAPK phosphatases (MKPs) are dual-specificity phosphatases (DUSPs) that dephosphorylate phosphothreonine and phosphotyrosine residues within MAPK^56^. We showed that ANGPTL1 stimulation increased the ERK1/2 phosphorylation in starved H295R cells. Based on this we suspected that there might be a connection between ANGPTL1, ERK1/2 phosphorylation and DUSP6. We found that ANGPTL1 treatment increased DUSP6 expression in starved H295R cells within 10 min (Fig. 8D) when ERK1/2 phosphorylation was fully stimulated (Fig. 8A). This result suggests that ANGPTL1 stimulates ERK phosphorylation and DUSP6 expression possibly for subsequent ERK dephosphorylation. On the other hand, it is known from the literature that ERK1/2 activation may induce phosphorylation of DUSP6 and its subsequent degradation by proteasomes^56^. However, we were not able to show an effect of ANGPTL1 on the steroid profile of H295R cells (data not shown). Thus, ANGPTL1 seems to modulate DUSP6 and ERK1/2 phosphorylation at a very sophisticated level to regulate CYP17A1 (Fig. 9).

## Discussion

In search of regulators of human androgen biosynthesis, we explored mechanisms underlying the induction of androgen production in serum starved H295R adrenal cells. The H295R cell model has been used in several other studies on androgen biosynthesis^11^,^57^,^58^. We have previously shown that serum starvation enhances the androgen production in H295R cells by increasing the CYP17A1-17,20-lyase activity and repressing HSD3B2 expression and activity^12^. This study provided us with a suitable model of a hyperandrogenic state which resembles adrenarche during human development.

In order to explore the mechanism behind starvation and search for new regulators of androgen biosynthesis we performed gene expression profiling under normal growth versus starvation growth conditions. Our results from gene expression profiling studies showed that fourteen genes were differentially expressed, three genes in the steroid biosynthetic process were directly affected and most of the other genes were involved in the energy metabolism and signal transduction, especially in the MAPKKK cascade. Gene enrichment analysis found an interesting network, suggesting that retinoic acid nuclear receptor RARB might have an indirect role in HSD3B2 regulation via Nur77.

It is evident from the literature that RA plays an important role in the regulation of steroidogenic gene transcription and steroid biosynthesis in many cells and tissues like the adrenal^42^, ovarian^39^, testicular^40^,^59^ and neural cells^41^. Retinoid receptors play a role in regulating StAR expression that is activated by RA^45^. Yet, the role of RARB in steroidogenesis remains unknown, which encouraged us to investigate its function in steroidogenesis. We now showed that RARB plays an important role in regulating StAR, HSD3B2 and CYP17A1 promoter activities and, in the presence of ATRA, the promoter activation was even more significant. Several published data have reported that ATRA regulates steroidogenic gene expression in different cell lines^39^,^40^,^41^. Various putative retinoic acid response elements have been identified in the StAR, HSD3B2, CYP17A1 and CYP11A1 promoters^60^.

To elucidate the role of RARB we overexpressed RARB in starved H295R cells, which showed that RARB was able to increase endogenous StAR expression. Interestingly, treatment with the RAR ligand ATRA lead to an increased StAR expression even when RARB was not overexpressed, suggesting that ATRA might regulate StAR expression by activating endogenous retinoid receptors like RARs and RXRs. This result is in line with a recently published work, which reported that ATRA regulates StAR expression by cooperation of both RARα and RXRα in MA-10 cells^45^. But for HSD3B2, we did not find any change after overexpression of RARB or ATRA treatments, suggesting that RARB might have indirect interactions with other receptors to regulate HSD3B2 expression. By contrast, steroid profiling revealed a significant effect of RARB and ATRA on androstenedione production in H295R cells suggesting enhanced HSD3B2 activity. In line with our finding, vitamin A deficient rats were found to resume androstenedione production when supplemented with retinol or RA^42^.

Gene enrichment analysis of our data suggested a role for RAR in regulating HSD3B2 through Nur77. RARB has been shown to interact with Nur77^46^, a well-known transcription factor for regulating HSD3B2^47^, therefore, we studied the co-operation between RARB and Nur77 in regulating HSD3B2. Under starvation condition, the transfection with Nur77 alone or in combination with RARB did not show any significant increase in HSD3B2 promoter activity. Under growth condition we found a significant increase in HSD3B2 promoter activity in cells transfected with Nur77 and RARB. These results suggest that Nur77 expression depends on the endogenous RARB expression. It is evident from chip data that RARB decreases under starvation which seems to affect Nur77 expression. Recent studies in human hepatocellular carcinoma cells showed that Nur77 and RARB interaction might stabilize Nur77 and sustain its expression level^46^.

Angiopoietin-like protein 1 (ANGPTL1) (also known as angiopoietin-related protein 1 (ARP1)) is the first member discovered of the ANGPTL family^52^. It is a secretory glycoprotein that is known to function as pro- as well as anti-angiogenic factor^49^,^50^. Our study demonstrated that ANGPTL1 induces ERK1/2 phosphorylation in starved H295R cells. This is in agreement with a recent publication showing that ANGPTL1 stimulates ERK phosphorylation in endothelial cells^50^. The ERK1/2 pathway is known to play major roles in cell proliferation, differentiation and survival^61^; therefore ANGPTL1 may have proliferative effects on starved H295R cells via activation of ERK1/2. Furthermore, we observed that ANGPTL1 stimulation leads to repressed CYP17A1 expression via induction of ERK phosphorylation. The effect of ERK phosphorylation on CYP17A1 expression has been reported in several studies^26^,^62^. Activation of ERK1/2 results in constitutive phosphorylation of SF-1 and subsequent decreased CYP17 expression^63^. In this study, we illustrate for the first time a role of ANGPTL1 in regulating CYP17A1 expression.

DUSP6 (also known as MKP3) is a cytoplasmic protein which is known to dephosphorylate specific ERKs^53^ that are members of the MAPK family. Our study revealed that ANGPTL1 stimulation alters DUSP6 expression in a time- and ERKs-dependent manner. On the other hand, it has been previously reported that ERK1/2 activation can induce phosphorylation of DUSP6 and its subsequent proteasomic degradation^56^. This suggests that ANGPTL1 induces DUSP6 and ERK phosphorylation which in turn control each other.

In initial experiments we also solved the questions whether the hyperandrogenic effect of starvation on steroidogenesis in H295R cells might be mediated through hormones or alterations in the cell cycle. Removal of hormones alone from the growth medium did not alter the steroid profile of H295R cells. Similarly, no involvement of the cell cycle was found using FACS analysis. Gene expression profiling revealed only one cell cycle related gene, PLK2 (a member of the polo like kinase family), which plays a role in normal cell division. But the significance of this gene in steroidogenesis is unknown. From that we concluded that neither hormones nor alterations in the cell cycle were key regulators of androgen production.

Finally, in our study we were also able to show that the hyperandrogenic effect of a starvation growth condition is not reserved to steroidogenesis of human adrenal H295R cells only. Mouse testis MA-10 cells likewise showed a marked induction of androstenedione production under starvation growth conditions. This finding suggests that the mechanisms involved in androgen regulation are neither species- nor tissue specific.

In conclusion, we gained further insight into the regulation of androgen production by studying starved, hyperandrogenic H295R cells. Neither hormones, nor cell cycle changes seemed to mediate the hyperandrogenic state in starved H295R cells. Only fourteen genes were differentially expressed in this condition. Transcription factor RARB was a key player in the regulatory network underlying starvation induced hyperandrogenism. RARB was an essential factor for regulating HSD3B2 through Nur77. We also established a role for the secretory protein ANGPTL1 in regulating steroidogenic CYP17A1 and DUSP6 expression through ERK1/2. Taken together, this study described new regulators of androgen biosynthesis in the starvation (hyperandrogenic) cell culture model.

## Methods

### Materials

Antibodies against phospho-ERK1/2, AKT, phospho-Akt (Ser473), DUSP6 were purchased from Cell Signaling Technology (Danvers, MA, USA). ERK1 (C-16) antibody was purchased from Santa Cruz Biotechnology (Dallas, TX, USA). Beta-actin antibody came from Sigma-Aldrich (St. Louis, MO, USA). Rabbit polyclonal antibody against human CYP17A1 was custom made by Genscript (Piscataway, NJ, USA)^12^. Chicken peptide antibody against human HSD3B2 was custom prepared by Genetel Labs (Madison, WI, USA) and anti-StAR antibody was kindly provided by Prof. Walter L. Miller (UCSF, San Francisco, CA, USA)^64^. Radioactive-labeled [7(*N*)-^3^H] pregnenolone (14 Ci/mmol) and [1,2,6,7(*N*)-^3^H] DHEA (63 Ci/mmol) were procured from PerkinElmer (Boston, MA, USA). All-trans-retinoic acid (ATRA), U0126 MEK inhibitor, charcoal, and dextran coated powder were purchased from Sigma-Aldrich. Propidium iodide was purchased from Becton Dickinson (BD) biosciences (Basel, Switzerland). ANGPTL1 recombinant protein was purchased from Abnova (Taipei City, Taiwan). Prof. Dr. Xiao-Kun Zhang, Sanford-Burnham Medical Research Institute (La Jolla, CA, USA) generously gave us the cDNA expression vector containing human RARB^65^. The -1.3kb StAR promoter reporter construct was kindly provided by Prof. Jerome F. Strauss III, VCU School of Medicine (Richmond, VA, USA)^66^. The -2327bp CYP11A1 promoter reporter construct was a gift from Prof. Walter L. Miller^9^. Other plasmids containing cDNAs and specific promoters were available in our laboratory from earlier projects^48^,^62^.

### Cell cultures and treatment

Human adrenocortical NCI-H295R cells were purchased from American Type Culture Collection (ATCC; CRL-2128). H295R cells were cultured in DMEM/Ham’s F-12 medium containing L-glutamine and 15 mM HEPES medium (GIBCO, Paisley, UK) supplemented with 5% NU-I serum (BD biosciences), 0.1% insulin, transferrin, and selenium (100 U/ml; GIBCO), penicillin (100 U/ml; GIBCO) and streptomycin (100 μg/ml; GIBCO). The serum free starvation medium consisted of DMEM/Ham’s F-12 medium, penicillin and streptomycin (100 μg/ml; GIBCO). Mouse Leydig (MA-10) cells were kindly provided by Prof. Brigitte M. Frey, Bern, Switzerland. MA-10 cells were cultured in Waymouth medium (Sigma–Aldrich) and supplemented with 15% horse serum (GIBCO), penicillin (100 U/ml; GIBCO) and streptomycin (100 μg/ml; GIBCO). For MA-10 cells, culture dishes were pre-coated with 0.1% gelatin (Sigma–Aldrich). The serum free starvation medium of MA-10 consisted of Waymouth medium and antibiotics. Charcoal treatment of NU-I serum was performed by adding charcoal-dextran coated powder (1 g) to NU-I serum (50 mL) while gently mixing on a shaker table overnight. The followingday, charcoal-stripped serum was obtained by centrifugation at 2’000 g for 15 minutes and filtration through a 0.2 μm filter.

### Cell cycle analysis

For cell cycle analysis H295R cells were cultured under normal growth and starvation conditions. After 48 h, cells were collected and 400 μl of Nicoletti-buffer (0.1% Na-citrate, 0.1% Triton X-100, and 50 μg/ml propidium iodide in PBS) was added to each sample in order to lyse the cell membranes and remove the cytoplasm. The propidium iodide stained DNA fragments were analyzed by flow cytometry (BD Biosciences). The quantification was done using FlowJo software (FlowJo, Ashland, OR, USA).

### Microarray analysis for gene expression profiling

Total RNA was isolated and purified with an RNeasy Micro kit (Qiagen GmbH, Hilden, Germany) from cultured H295R cells under normal growth and serum starvation conditions. Microarray experiments were carried out from three independent experiments with GeneChip Human Gene 1.0 ST arrays (Affymetrix Inc., Santa Clara, CA, USA) at Genomic Technologies Facility (GTF) of the University of Lausanne, Switzerland. The data were analyzed using Affymetrix Power Tools package (for 1.0-ST arrays). All statistical analyses were performed using the free high-level interpreted statistical language R and various Bioconductor packages (http://www.Bioconductor.org). The *P* values were adjusted for multiple testing with Benjamini and Hochberg’s method to control for (any or the) false discovery rate (FDR)^67^. Probe sets showing at least a 2-fold change and an FDR<0.05 were considered significant. Hierarchical clustering was carried out using Cluster 3.0 and JTreeView software^68^,^69^. Differentially expressed genes were analyzed to identify their involvement in specific biological processes, pathways or networks using the software Database for Annotation, Visualization and Integrated Discovery (DAVID) v6.5 (http://david.abcc.ncifcrf.gov)^38^,^70^ and GeneGo MetaCore analysis software (GeneGo Inc., Minneapolis, MN, USA).

### Quantitative real time PCR (qRT-PCR)

H295R cells were cultured under normal growth and starvation conditions and total RNA was isolated using the TRIzol method according to the manufacturer’s instructions (Invitrogen Life Technologies, Carlsbad, CA, USA). RNA was reverse-transcribed to cDNA using the Improm RNA transcriptase kit (Promega) as previously described^12^,^71^. qRT-PCR analysis was performed on the 7500-Fast real-time PCR System (Applied Biosystems, Foster City, CA, USA) using ABsolute SYBR Green Mix (ABgene; Thermo Fisher scientific, Waltham, MA, USA). Briefly, qRT-PCR was performed in 96 well plates using 50 ng/well cDNA and 1 μl (20 pmol/μl) specific primers (Microsynth, Balgach, Switzerland) in a total volume of 25 μl. The cyclophilin A gene was used as endogenous control. Specific primer sequences may be found in Supplementary Table S1. Fold change in gene expression for a particular gene was calculated by the 2^−ΔΔCt^ method^72^,^73^. Amplification curves and the mean cycle threshold (Ct) values were calculated using the 7500 Fast System SDS software (Applied Biosystems), and correction for the endogenous gene, ΔCt and ΔΔCt were calculated as previously described^12^.

### Cell transfection and dual luciferase promoter assays

H295R cells were transfected with the empty vector (pGL3, Δluc) or specific promoter-reporter constructs (−3.7 kb CYP17A1, −1.05 kb HSD3B2, –1.3 kb StAR, -2327 bp CYP11A1). Transient transfection was carried out in 24-well plates for 6 h (Falcon 3047; Becton Dickinson) using Lipofectamine 2000 reagent (Invitrogen). Per 24-well, the transfection mixture contained 0.5 μg promoter plasmid DNA (StAR, HSD3B2, CYP17A1 and CYP11A1) and 125 ng coregulator plasmid cDNA (e.g. RARB and Nur77) as well as 25 ng Renilla luciferase reporter (pRL-TK) (Promega, Maidson, WI) for endogenous control. Transfection medium [DMEM containing L-glutamine and 15 mm HEPES (GIBCO) supplemented with 10% NU-I serum (BD biosciences) and 0.1% selenium/insulin/transferrin (GIBCO)] was changed after 6 h and cells were cultivated in serum free medium for 24 h. Thereafter, cells were grown in the presence or absence of 1 μM ΑΤRA in serum-free medium for another 24 h. Finally, 48 hours after transfection, cells were washed with phosphate buffered saline (PBS) and then assayed for luciferase activity using the Dual Luciferase Reporter Assay system and the protocol by Promega. For overexpression studies of RARB, H295R cells were either transfected with the empty vector (pCMV5) or the vector expressing the cDNA of RARB in six wells (1.0 × 10^6^ cells per well; Falcon 3046;BD biosciences) using Lipofectamine 2000 (Invitrogen).

### Western blot analysis

For protein analyses, treated cells were washed with ice-cold PBS and harvested in lysis buffer. Protein extraction and concentration measurement were done as previously described^48^. For Western blots, 30 μg protein of total cell lysates was mixed with SDS loading buffer (62.5 mM Tris–HCl, pH 6.8; 2% sodium dodecylsulfate, 10% glycerol, 100 mM dithiotreitol, 0.01% bromophenol blue), heated for 5 min at 95 °C, separated on a 10% SDS-PAGE gel and blotted on an Immobilon-FL PVDF transfer membrane (Millipore, Billerica, MA, USA) using the semi-dry transfer method. Blots were blocked for 1 h with 5% non-fat dry milk or with Odyssey blocking buffer (LI-COR Bioscience Inc., Lincoln, NE, USA). Then the blots were incubated overnight at 4 °C with primary antibodies under specific conditions as follows: HSD3B2, 1:10′000 in 5%Milk/TTBS, CYP17A1 and StAR, 1:5’000 in 0.1% Tween20/Odyssey blocking buffer, phospho-ERK1/2, phospho-AKT, AKT, ERK 1:1’000 in 3% BSA/TTBS. For β-actin, blots were blocked in 5% non-fat dry milk for 1 h at room temperature and incubated in 1% BSA/TTBS for 1 h with a mouse anti-β-actin antibody. The following secondary antibodies were used 1:15’000 at room temperature for 1 h in 3% milk or in Odyssey blocking buffer: IRDye 800CW donkey anti-rabbit, IRDye 800CW donkey anti-chicken and IRDye 680RD goat anti-mouse (LI-COR Bioscience Inc.). After the final washes, the membranes were scanned and the signals of reactive bands were quantified using the Odyssey Sa Infrared Imaging system (LI-COR Bioscience Inc.).

### Steroid profiling

Steroid profiling was done from cell cultures on 6 well plates by adding 100,000 cpm [^3^H] pregnenolone to the culture medium for 90 min. Steroids were extracted from cell supernatants and separated by thin layer chromatography (TLC) as previously described^12^,^48^,^71^. Steroid conversion was assessed as a percentage of incorporated radioactivity into a specific steroid product in relation to total radioactivity measured for the whole sample (internal control). To confirm the identified steroids from TLC profiles, we were running control experiments for both H295R and MA-10 cells assessing the steroids by gas chromatography mass spectrometry (data not shown).

### Statistical analysis

Statistical analysis was performed with Microsoft Excel and software Prism 6 (Graph Pad Software, Inc. San Diego, CA, USA). Statistical differences between values were calculated using the Student’s t test or the two-way ANOVA analysis as appropriate. Quantitative data represent the mean of at least three independent experiments, error bars indicate the mean ± SD. Significance was set at *p < 0.05 and **p < 0.01.

## Additional Information

**How to cite this article**: Udhane, S. S. *et al*. Retinoic acid receptor beta and angiopoietin-like protein 1 are involved in the regulation of human androgen biosynthesis. *Sci. Rep.* **5**, 10132; doi: 10.1038/srep10132 (2015).

## Supplementary Material

Supplementary Information

## Figures and Tables

**Table 1:** List of differentially expressed genes in H295R cells under normal growth vs starvation conditions.

**Order**	**Accession Number**	**Gene Symbol**	**Gene Title**	**Fold Change**
**1**	NM_000198	HSD3B2	3 beta-hydroxysteroid dehydrogenase type 2	3.97
**2**	NM_000965	RARB	Retinoic acid receptor, beta	2.53
**3**	NM_004673	ANGPTL1	Angiopoietin-like 1	3.30
**4**	NM_006622	PLK2	Polo-like kinase 2	2.62
**5**	NM_001946	DUSP6	Dual specificity phosphatase 6	2.02
**6**	NM_007207	DUSP10	Dual specificity phosphatase 10	2.29
**7**	NM_002523	NPTX2	Neuronal pentraxin II	2.00
**8**	NM_000050	ASS1	Argininosuccinate synthase 1	2.06
**9**	NM_003633	ENC1	Ectodermal-neural cortex 1	2.21
**10**	NM_000204	CFI	Complement factor I	2.09
**11**	NM_031455	CCDC3	Colied-coil domain containing 3	2.58
**12**	NM_207361	FREM2	FRAS1 related extracellular matrix protein 2	2.04
**13**	NM_004316	ASCL1	Achaete-scute complex homolog 1	−2.09*
**14**	NM_000862	HSD3B1	3 beta-hydroxysteroid dehydrogenase type 1	2.48

We performed microarray studies using the GeneChip Human Gene 1.0 ST array. For analysis, the *P* values were adjusted for multiple testing with Benjamini and Hochberg’s method to control for false discovery rate (FDR). Probe sets showing at least a 2-fold change and an FDR < 0.05 were considered significant. We identified 14 genes with a significantly altered expression profile when comparing starved with control H295R cells.

^*^Sign indicates up-regulation.

**Table 2:** Suggested biological function of the differentially expressed genes under starvation.

**Biological Function**	**Overexpressed**	**Underexpressed**
Steroid biosynthetic process		HSD3B2, HSD3B1, CYP21A2
MAPKKK cascade		DUSP6, DUSP10
Signal transduction		PLK2, DUSP6, DUSP10, FREM2, ANGPTL1
Metabolic process	ASCL1	ASS1, CFI, DUSP10, DUSP6, ENC1, PLK2, RARB, HSD3B2, HSD3B1

Differentially expressed genes in normal growth vs starvation conditions were loaded into DAVID v6.5 software to perform biological functional analysis. Analysis was performed on the 14 gene transcripts identified by microarray analysis setting the fold change cut-off at 2.0 with an adjusted p-value < 0.05.
