# Supplementary material for: Retinoic acid receptor beta and angiopoietin-like protein 1 are involved in the regulation of human androgen biosynthesis
Source: Sci Rep. 2015 May 13;5:10132. doi: 10.1038/srep10132 (PMC4429542; doi:10.1038/srep10132)
Supplement: Supplementary Information [file srep10132-s1.pdf]

Supplementary Information

Manuscript: Retinoic acid receptor beta and angiopoietin-like protein 1 are involved in the regulation of human androgen biosynthesis

Authors: Sameer S. Udhane, Amit V. Pandey, Gaby Hofer, Primus E. Mullis, and Christa E. Flück

Supplementary Table S1. List of primer sequences used for qRT-PCR
GeneBank accession numbers for source sequences are given in parentheses.

**Primers for qRT-PCR**	
**HSD3B2 (NM_000198)**	S: 5’- AAGCTGACTGTACTTGAAGG – 3’
	AS: 5’- GTGTACAAGGTATCACCATT – 3’
**RARB (NM_000965)**	S: 5’- GGTTTCACTGGCTTGACCAT – 3’
	AS: 5’- GGCAAAGGTGAACACAAGGT – 3’
**ANGPTL1 (NM_004673)**	S: 5’- CACCACCTGATCTGGCAACT – 3’
	AS: 5’- AATCCCCTGCATTTCCCTGG – 3’
**PLK2 (NM_006622)**	S: 5’- CCACCATCACCACCATTCGC – 3’
	AS: 5’- CAGACACAATCTGCCTGAGGT – 3’
**DUSP6 (NM_001946)**	S: 5’- GTTTTTCCCTGAGGCCATTTC – 3’
	AS: 5’- TCACAGTGACTGAGCGGCTAAT – 3’
**DUSP10 (NM_007207)**	S: 5’- CTGAACATCGGCTACG – 3’
	AS: 5’- GGTGTAAGGATTCTCGGT – 3’
**NPTX2 (NM_002523)**	S: 5’- CATCGAGCTGCTCATCAAC – 3
	AS: 5’- CTGCTCTTGTCCAAGGATC – 3’
**ASS1 (NM_000050)**	S: 5’- GCCCGCAAACAAGTGGAAAT – 3’
	AS: 5’- TCCAGGATTCCAGCCTCGTA – 3’
**ENC1 (NM_001256576)**	S: 5’- GTCCTTCTCCATGCCGGAAA – 3’
	AS: 5’- GGGCAGCTGGAGGAAATCTT – 3’
**CFI (NM_000204)**	S: 5’- GCCATGGCAGAGATGCATTG – 3’
	AS: 5’- GAAGCCTTTGCCTTGGCATG – 3’
**CCDC3 (NM_031455)**	S: 5’- GGGCTACTTCTCGTGCCACTC – 3’
	AS: 5’- TGCCGGTTGCGCTTCTCC – 3’
**FREM2 (NM_207361)**	S: 5’- AGTCGCCACTGTTGAGATCG – 3
	AS: 5’- ACATCTCCGCTCCTCCTGAT – 3’
**ASCL1 (NM_004316)**	S: 5’- GCGGCCAACAAGAAGATG – 3’
	AS: 5’- CAAGTCGTTGGAGTAGTTGG – 3’
**CYP21A2 (NM_000500)**	S: 5’- AGAGGGATCACATCGTGGAG – 3’
	AS: 5’- CTTCCAGGAGCTGTCCAGAG – 3’
**Cyclophilin A (NM_021130)**	S: 5’- GGCAAATGCTGGACCCAACACA – 3’
	AS: 5’- TGCTGGTCTTGCCATTCCTGGA – 3’

Supplementary Table S2. List of differentially expressed genes in H295R cells under normal growth vs starvation conditions (>1.5 fold)

By GeneChip Human Gene 1.0 ST array studies. We identified 63 genes when setting the fold change cut-off at 1.5 with an adjusted p-value < 0.05.

Order	Accession Number	Gene Symbol	Gene Title	Fold Change
**1**	NM_000500	CYP21A2	Cytochrome P450, family 21, subfamily A, polypeptide 2	1.83523
**2**	NM_170744	UNC5B	Unc-5 homolog B	1.86973
**3**	NM_003009	SEPW1	Selenoprotein W, 1	1.96531
**4**	NM_001017425	KCNK2	Potassium channel, subfamily K, member 2	-1.7602*
**5**	NM_020338	ZMIZ1	Zinc finger, MIZ-type containing 1	1.64539
**6**	NM_014848	SV2B	Synaptic vesicle glycoprotein 2B	-1.6558*
**7**	NM_016522	NTM	Neurotrimin	-1.6963*
**8**	NM_001197294	DPYSL3	Dihydropyrimidinase-like 3	1.7987
**9**	NM_000624	SERPINA5	Serpin peptidase inhibitor, clade A (alpha-1 antiproteinase, antitrypsin), member 5	1.5313
**10**	NM_001553	IGFBP7	Insulin-like growth factor binding protein 7	1.77783
**11**	NM_005314	GRPR	Gastrin-releasing peptide receptor	-1.8474*
**12**	NM_002514	NOV	Nephroblastoma overexpressed gene	1.8505
**13**	NM_001490	GCNT1	Glucosaminyl (N-acetyl) transferase 1, core 2	1.92664
**14**	NM_001128176	THRB	Thyroid hormone receptor, beta	1.67774
**15**	NM_018407	LAPTM4B	Lysosomal protein transmembrane 4 beta	1.86459
**16**	NM_006084	IRF9	Interferon regulatory factor 9	1.76116
**17**	NM_016546	C1RL	Complement component 1, r subcomponent-like	1.53157
**18**	NM_001874	CPM	Carboxypeptidase M	-1.7492*
**19**	NM_002934	RNASE2	Ribonuclease, RNase A family, 2 (liver, eosinophil-derived neurotoxin)	1.7072
**20**	NM_021202	TP53INP2	Tumor protein p53 inducible nuclear protein 2	1.6294
**21**	NM_030762	BHLHE41	Basic helix-loop-helix family, member e41	1.6084
**22**	NM_004454	ETV5	Ets variant 5	1.82798
**23**	NM_201555	FHL2	Four and a half LIM domains 2	1.53679
**24**	NM_003810	TNFSF10	Tumor necrosis factor (ligand) superfamily, member 10	1.61757
**25**	NM_173582	PGM2L1	Phosphoglucomutase 2-like 1	1.69809
**26**	NM_004460	FAP	Fibroblast activation protein, alpha	1.88712
**27**	NR_024101	FLJ35776	Hypothetical LOC649446	1.64384
**28**	NM_007053	CD160	CD160 molecule	1.67532
**29**	NM_007361	NID2	Nidogen 2 (osteonidogen)	-1.5459*
**30**	NM_201524	GPR56	G protein-coupled receptor 56	1.71853
**31**	NM_021643	TRIB2	Tribbles homolog 2 (Drosophila)	1.5145
**32**	NM_003048	SLC9A2	Solute carrier family 9 (sodium/hydrogen exchanger), member 2	-1.5989*
**33**	NM_005923	MAP3K5	Mitogen-activated protein kinase kinase kinase 5	1.71674
**34**	NM_020809	ARHGAP20	Rho GTPase activating protein 20	1.5001

**35**	NM_001771	CD22	CD22 molecule	1.70581
**36**	NM_004172	SLC1A3	Solute carrier family 1 (glial high affinity glutamate transporter), member 3	-1.5469*
**37**	NM_133445	GRIN3A	Glutamate receptor, ionotropic, N-methyl-D-aspartate 3A	-1.5509*
**38**	NM_001195032	C6orf99	Chromosome 6 open reading frame 99	-1.6939*
**39**	NM_002543	OLR1	Oxidized low density lipoprotein (lectin-like) receptor 1	1.52082
**40**	NM_153366	SVEP1	Sushi, von Willebrand factor type A, EGF and pentraxin domain containing 1	1.86166
**41**	NM_152637	METTL7B	Methyltransferase like 7B	1.67562
**42**	NM_005810	KLRG1	Killer cell lectin-like receptor subfamily G, member 1	1.66086
**43**	NM_024704	KIF16B	Kinesin family member 16B	1.64102
**44**	NM_015236	LPHN3	Latrophilin 3	1.58606
**45**	NM_002298	LCP1	Lymphocyte cytosolic protein 1 (L-plastin)	-1.5299*
**46**	NM_022843	PCDH20	Protocadherin 20	1.50787
**47**	NM_001013625	C1orf192	Chromosome 1 open reading frame 192	-1.5502*
**48**	NM_199511	CCDC80	Coiled-coil domain containing 80	1.57305
**49**	NM_012281	KCND2	Potassium voltage-gated channel, Shal-related subfamily, member 2	-1.5775*
**50**	NM_015441	OLFML2B	Olfactomedin-like 2B	1.639
**51**	NM_014705	DOCK4	Dedicator of cytokinesis 4	1.56717
**52**	NM_018672	ABCA5	ATP-binding cassette, sub-family A (ABC1), member 5	1.64576
**53**	NM_170672	RASGRP3	RAS guanyl releasing protein 3 (calcium and DAG-regulated)	1.5215
**54**	NM_001128596	TC2N	Tandem C2 domains, nuclear	1.50841
**55**	NM_212482	FN1	Fibronectin 1	1.59342
**56**	NM_001161429	RANBP3L	RAN binding protein 3-like	1.60935
**57**	NM_002164	IDO1	Indoleamine 2,3-dioxygenase 1	1.61038
**58**	NR_003530	MEG3	Maternally expressed 3 (non-protein coding)	1.64795
**59**	NM_001099694	ZNF578	Zinc finger protein 578	-1.7452*
**60**	NM_001012980	SATL1	Spermidine/spermine N1-acetyl transferase-like 1	-1.5217*
**61**	NM_001102562	MARCH11	Membrane-associated ring finger (C3HC4) 11	-1.5395*
**62**	NR_003231	SNORD113-3	Small nucleolar RNA, C/D box 113-3	1.56858
**63**	NR_003194	SNORD114-2	Small nucleolar RNA, C/D box 114-2	1.55025

*- Sign indicates up-regulation

Supplementary Table S3. Suggested biological function of the 63 genes found differentially expressed at fold change > 1.5

Differentially expressed genes in normal growth vs starvation conditions were loaded into DAVID v6.5 software to perform biological functional analysis. Analysis was performed on the 63 gene transcripts identified by microarray analysis setting the fold change cut-off at 1.5 with an adjusted p-value < 0.05.

Biological Function	Overexpressed	Under expressed
**Steroid biosynthetic process**		CYP21A2
**MAPKKK cascade**		RASGRP3, MAP3K5
**Signal transduction**	GRPR, GRIN3A, SLC1A3	CD22, GPR56, RASGRP3, ARHGAP20, DOCK4, FAP, FN1, LPHN3, NOV, OLFML2B, PCDH20, THRB, TRIB2, TNFSF10, UNC5B
**Cell adhesion**	NTM, NID2	CD22, GPR56, FN1, IGFBP7, NID2, OLR1, PCDH20, SVEP1
**Inflammatory response**		C1RL, CFI, FN1, IDO1, KLRG1, OLR1
**Glycosylation process**	CPM, GRPR, GRIN3A, NID2, NTM, KCNK2, SLC9A2, SV2B	ABCA5, CD160, CD22, GPR56, PCDH20, C1RL, FN1, GCNT1, IGFBP7, KLRG1, LPHN3, NOV, OLFML2B, OLR1, RNASE2, SERPINA5, SVEP1, UNCB

Supplementary Figure S1. Heat Map of Affymetrix microarray data showing differentially expressed genes in H295R cells grown under normal and starvation conditions.

H295R cells were grown under serum-free, starvation (SM) or normal (GM) conditions. RNA was extracted and analyzed for differential gene expression by Affymetrix GeneChip Human Gene 1.0 ST arrays. Data were then analyzed by Cluster 3.0 and Jtreeview software for creating a representative heat map of the 14 genes which were identified at a significance level of fold change > 2. In the heat map graphic rows represent the individual genes. The gene expression levels are displayed for each independent sample. Both genes and samples were clustered by a complete linkage method**.** Over-expression is indicated in red, under-expression in green.

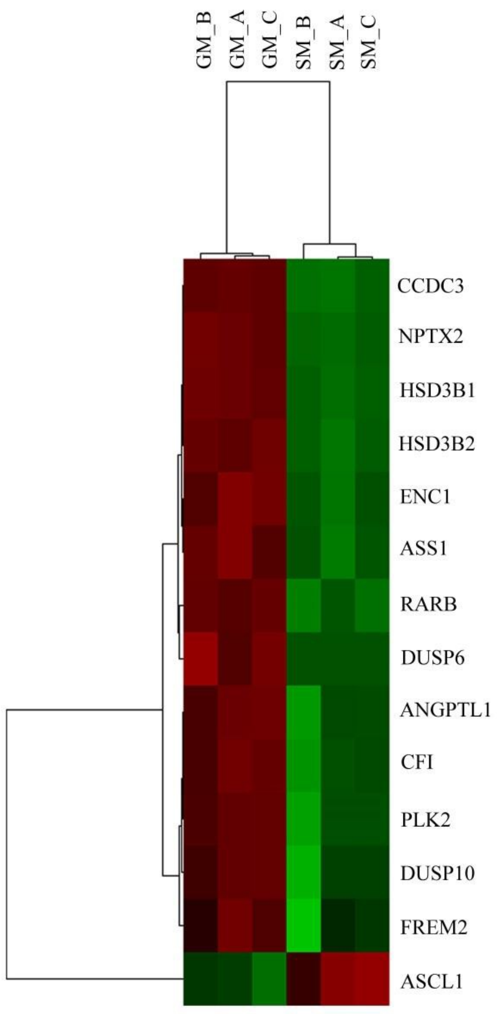

Supplementary Figure S1

Supplementary Figure S2. Effect of ATRA dose response on H295R steroid profile.

H295R cells were cultivated in serum free medium for 24 h. Thereafter, cells were grown in presence or absence of ATRA in serum-free medium for another 24 h. Steroid production was labeled with [^{3}H] pregnenolone for 90 min. Steroids were extracted and resolved by TLC. A representative TLC is shown on the left and quantitative analysis of steroids on the right side. We found ATRA treatment significantly altered the steroid production when compared to the control. Data are the mean ± SEM of two independent experiments. * p<0.05, ** p<0.01.

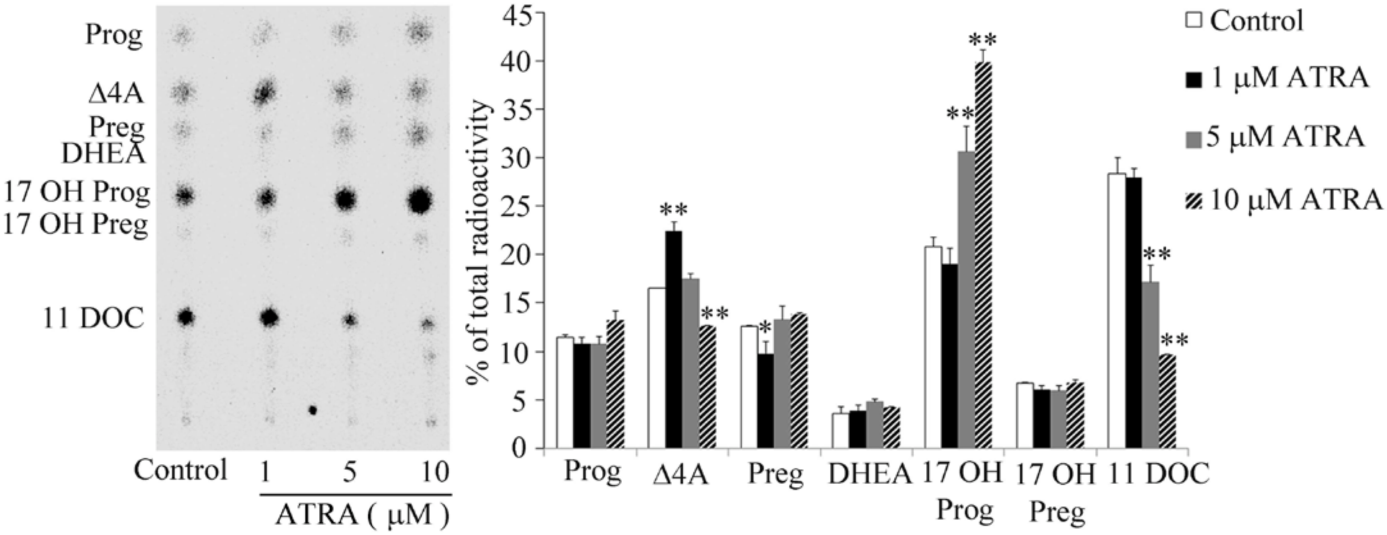

Spplementary Figure S2
